# Lisinopril Mitigates Radiation-Induced Mitochondrial Defects in Rat Heart and Blood Cells

**DOI:** 10.3389/fonc.2022.828177

**Published:** 2022-03-02

**Authors:** Saryleine Ortiz de Choudens, Rodney Sparapani, Jayashree Narayanan, Nicole Lohr, Feng Gao, Brian L. Fish, Monika Zielonka, Tracy Gasperetti, Dana Veley, Andreas Beyer, Jessica Olson, Elizabeth R. Jacobs, Meetha Medhora

**Affiliations:** ^1^ Department of Radiation Oncology, Froedtert & the Medical College of Wisconsin, Milwaukee WI, United States; ^2^ Institute for Health and Equity, Medical College of Wisconsin, Milwaukee, WI, United States; ^3^ Cardiovascular Center, Froedtert & the Medical College of Wisconsin, Milwaukee, WI, United States; ^4^ Cancer Center, Froedtert & the Medical College of Wisconsin, Milwaukee, WI, United States; ^5^ Department of Biophysics, Medical College of Wisconsin, Milwaukee, WI, United States; ^6^ Department of Physiology, Medical College of Wisconsin, Milwaukee, WI, United States; ^7^ Department of Pulmonary Medicine, Froedtert & the Medical College of Wisconsin, Milwaukee, WI, United States; ^8^ Department of Research Service, Veterans Affairs, Zablocki VA Medical Center (VAMC), Milwaukee, WI, United States

**Keywords:** cardiotoxicity, thoracic radiation, mitochondrial dysfunction, rat model, lisinopril

## Abstract

The genetic bases and disparate responses to radiotherapy are poorly understood, especially for cardiotoxicity resulting from treatment of thoracic tumors. Preclinical animal models such as the Dahl salt-sensitive (SS) rat can serve as a surrogate model for salt-sensitive low renin hypertension, common to African Americans, where aldosterone contributes to hypertension-related alterations of peripheral vascular and renal vascular function. Brown Norway (BN) rats, in comparison, are a normotensive control group, while consomic SSBN6 with substitution of rat chromosome 6 (homologous to human chromosome 14) on an SS background manifests cardioprotection and mitochondrial preservation to SS rats after injury. In this study, 2 groups from each of the 3 rat strains had their hearts irradiated (8 Gy X 5 fractions). One irradiated group was treated with the ACE-inhibitor lisinopril, and a separate group in each strain served as nonirradiated controls. Radiation reduced cardiac end diastolic volume by 9-11% and increased thickness of the interventricular septum (11-16%) and left ventricular posterior wall (14-15%) in all 3 strains (5-10 rats/group) after 120 days. Lisinopril mitigated the increase in posterior wall thickness. Mitochondrial function was measured by the Seahorse Cell Mitochondrial Stress test in peripheral blood mononuclear cells (PBMC) at 90 days. Radiation did not alter mitochondrial respiration in PBMC from BN or SSBN6. However, maximal mitochondrial respiration and spare capacity were reduced by radiation in PBMC from SS rats (p=0.016 and 0.002 respectively, 9-10 rats/group) and this effect was mitigated by lisinopril (p=0.04 and 0.023 respectively, 9-10 rats/group). Taken together, these results indicate injury to the heart by radiation in all 3 strains of rats, although the SS rats had greater susceptibility for mitochondrial dysfunction. Lisinopril mitigated injury independent of genetic background.

## Introduction

Breast and lung cancer accounts for 28% of new cancer diagnoses in the United States, and 29% of cancer deaths (1). Radiation therapy is an essential part of treatment for these malignancies, with more than 50% of patients receiving radiation (2). Exposure of radiation to thoracic structures can cause a wide variety of acute symptoms and delayed toxicities, including cardiac injuries (3). Radiation to the heart is often unavoidable when treating lung, breast, esophageal, and other thoracic malignancies. Efforts to increase treatment doses are limited by normal tissue tolerance. With the increasing role of radiation therapy in the contemporary treatment of cancer, patients that are long term survivors are at risk of cardiovascular injury and mortality (2, 3). High-dose radiation exposure to the heart can cause cardiac dysfunction, developing months to decades following treatment (4, 5). This includes injury to the cardiac tissues and vasculature, which can lead to complications such as pericarditis, coronary artery disease, ischemic heart disease, congestive heart failure, conduction defects and valvular dysfunction (6, 7).

One of the features of cardiac injury is the formation of fibrosis, distinguished by collagen deposition both inside and surrounding cardiomyocytes (4, 7–9). Radiation induced cardiotoxicity can range from issues with contractility, nerve impulse transmission, fibrosis, and compliance, resulting in arrhythmia and heart failure. Additional causes of cardiac injury include endothelial cell damage and activation of inflammatory and atherosclerotic responses (4, 7, 8, 10–15). When examining heart and lung irradiation in rats, Ghobadi et al. (16) concluded that combined irradiation of lung and heart induced pronounced increases in left ventricular end-diastolic pressure and relaxation time, in addition to an increase in right ventricle end-diastolic pressure, indicative of biventricular diastolic dysfunction (16).

Advances in imaging and radiotherapy delivery techniques have helped to reduce cardiac exposure (17–22). However, there is no known safe dose for cardiac exposure, and heart radiation exposure often remains unavoidable (4). The underlying causes and biomarkers of radiation-induced cardiotoxicity are currently unknown, prompting the need for experimental models with inherent differences in sensitivity and resistance to the development of radiation-induced cardiotoxicity (19). There have been numerous preclinical cell and animal models that have been used to study the mechanisms behind radiation-induced heart dysfunction (2, 4). Nonetheless, the mechanism of cardiac injury has not yet been fully elucidated (4). By improving our understanding of the biological pathways and mechanisms involved in radiation induced normal tissue toxicity, cancer treatment can be improved, with the goal of achieving maximum therapeutic benefit and reduced toxicities (2).

Preclinical rat models have been used to decipher genetic and molecular regulation of radiation-induced injury to normal tissues. For example, Dahl salt-sensitive rats (SS rats) develop hypertension and related cardiovascular and kidney diseases when fed a high salt diet (23, 24). The SS rats also exhibit early onset renal dysfunction (25, 26), with low renin activity, as compared to non-salt sensitive strains such as Brown Norway rats (BN rats). Both these strains have been used previously to identify genetic modifiers of radiation induced cardiotoxicity (19). A consomic strain SSBN3, with substitution of rat chromosome 3 from the BN rat strain into the SS background has also been used. Radiation induced cardiotoxicity was more severe in SS rats as compared to BN or SSBN3 rats without altering levels of dietary salt intake. In the current study, we use SS and BN rats along with SSBN6 rats that have a substitution of rat chromosome 6 from the resistant BN strain into the SS background to further explore genetic diversity in radiation sensitivity (27). Such chromosome substitution strains (consomics) have been commonly used to identify genetic loci that modulate response (19). Chromosome 6 from BN to the SS background conferred mitochondrial preservation and cardioprotection during *ex vivo* myocardial ischemia reperfusion (27). Because 73% of hypertensive and 36% of normotensive African Americans have salt sensitivity and low renin activity, compared to 56% of White hypertensive individuals (24–26, 28), SS-rats have been used as surrogates to study hypertension and kidney diseases common to African Americans (24–26). Evidence suggests that the proinflammatory effects of aldosterone contribute to both hypertension and to hypertension-related vascular disease (29). Sensitivity to radiation has not been well studied in diverse populations including African Americans, underscoring the usefulness of the SS rats as models.

The heart is made up of cells that are enriched in mitochondria, which are the powerhouse for myocytes. Mitochondrial derived ATP *via* oxs-phos is a hallmark of physiological cardiac function. With beginning of disease, cardiac metabolism changes to glycolysis in part due to mtDNA damage (30). Under pathological conditions, mitochondria are also a major source of reactive oxygen species, which has been reviewed by Stowe and Camara (31), including exposure to ionizing radiation (32). Radiation injury is mediated by DNA damage, so that extranuclear mitochondrial DNA is an important target. Mitochondria account for up to 30% or more of the cell volume in the heart and certain blood cells (32). Immediately after radiation, transient oxidative stress is generated by radiolysis of water (32, 33), but following that, oxidative stress from intracellular activities such as mitochondrial electron transport systems add to the delayed effects of radiation. Mutations in mitochondrial DNA, as well as changes in intracellular cytokine and signaling cascades induced by radiation, generate waves of oxidative stress that lead to cell death and apoptosis. This pathological process can continue for months post-irradiation (32). Partial deactivation of mitochondrial respiratory complexes have been reported in irradiated mouse hearts weeks after irradiation (34). More recently, cellular oxidative stress has been measured by the ‘Bioenergetic Health Index (BHI)’ (35). Using high-throughput assays to measure oxygen consumption, cellular bioenergetics are described to serve as a sensitive biomarker of health. Changes such as mitochondrial dysfunction have been reported in diabetes, cardiovascular disease, cancer, and toxic chemical exposures (36). Interestingly, mitochondrial activity in cells throughout the body was found to be altered by diseases associated with a specific organ, including diabetes and neurodegeneration (36–39). Since circulating peripheral blood mononuclear cells (PBMC) are easily accessible, the BHI in PBMC could potentially serve as a biomarker of organ diseases, such as cardiotoxicity, after localized radiation to the heart and lung.

Angiotensin-converting enzyme inhibitors have been found to mitigate many of the delayed injuries to cardiac and pulmonary systems (16, 40), and decrease functional and structural damage in irradiated hearts (41). In humans, ACE inhibitors are known to protect the heart from remodeling by reducing the effects of angiotensin II (42–45). We hypothesize that radiation to the heart will cause cardiac injury that will be evidenced by functional changes (monitored by echocardiogram) in our rat models. This injury will be reduced by the addition of ACE inhibitors. Using the changes in echocardiogram parameters we aimed to identify functional changes that occurred in the hearts of irradiated SS, BN and SSBN6 rats, as well as to determine if the changes in mitochondrial bioenergetics in PBMC could serve as a relatively non-invasive biomarker to predict genetically regulated, disparate responses to radiation.

## Materials and Methods

### Animal Models

Small animal models, including rodents, have been used for many decades to study cardiac radiation toxicity, given the physiological similarities that these models have to humans. In order to mirror the radiation doses and fractionations delivered to humans as part of their cancer treatment, we used fractionated radiation including the left lung and heart. To target these organs accurately, image guided radiation was used. The X-Rad SmART research platform (Precision X-ray) is a CBCT image guided system, that allows for irradiation of small experimental animals, and can provide an accuracy that is similar to clinical RT (2, 46).

Three animal models were used for this project. All rats were generated, bred and genotyped in the Medical College of Wisconsin’s Genomic Sciences & Precision Medicine Center. Brown Norway (BN) rats were used as the control group to model a radiation resistant strain of rats. Salt Sensitive (SS) rats represent a low renin model that is more likely to be hypertensive. The third group are the SSBN6, which are SS rats with substitution of rat chromosome 6 from the resistant BN rat strain into the SS background, with the goal of attenuating radiation induced cardiotoxicity (27). Using chromosome substitution strains (consomics) has been a strategy used to identify complex genetic modifiers of cardiovascular phenotypes (19). All rats were female because of greater background information regarding response to heart and thoracic irradiation than in males (40, 47). For the BN rat model, 10 rats were assigned to the control group, 10 rats received radiation, 5 rats received lisinopril and 5 rats received both radiation and lisinopril. For the SS rat model, 10 rats were assigned to the control group, 10 rats received radiation, 10 rats received lisinopril and 10 rats received both radiation and lisinopril. For the SSBN6 rats, 5 rats were assigned to the control group, 5 rats received radiation, 3 rats received lisinopril and 5 rats received both radiation and lisinopril.

### Animal Care

All procedures in this study were performed according to the American Guidelines for the Ethical Care of Animals and approved by the Institutional Animal Care and Use Committee of the Medical College of Wisconsin.

### Animals and Irradiation

**Figure 1:**
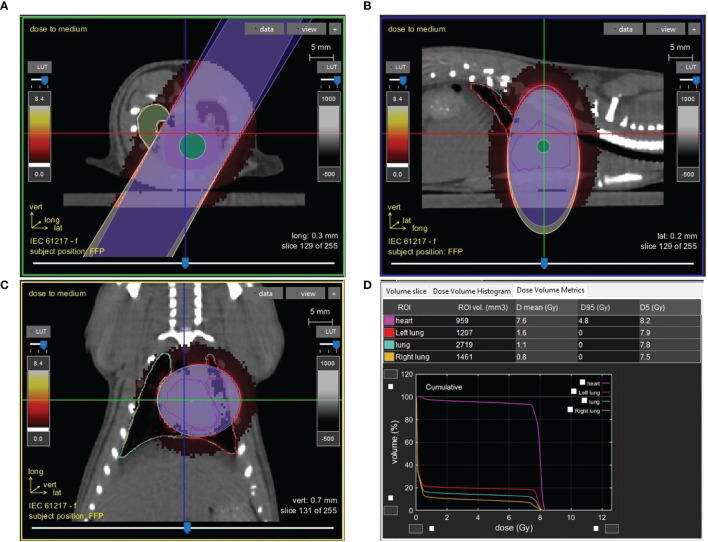
Image guided cardiac irradiation computed tomography images of a representative female rat with a 1.5cm diameter circular collimator plan with radiation dose to isocenter of 8 Gy × 5 fractions with equally weighted parallel opposed beams are shown in the axial **(A)**, sagittal **(B)**, and coronal **(C)** planes. Panel **(D)** shows the dose volume histogram and metrics demonstrating dose to the heart, left lung and right lung.

SS, BN, and SSBN consomic SSBN6 rats (Medical College of Wisconsin), aged 11 to 13 wk, were randomly allocated to different treatment groups. Left thoracic irradiation was performed using the high-precision image-guided SmART irradiator (Precision X-Ray, North Branford, CT). Rats were anesthetized by 3% isoflurane/room temperature air inhalation for the duration of each treatment. Pilot V1.8 Imaging Software (University Health Network, Toronto, Canada) was used to create two-dimensional projections over 360° to provide computed tomography scans in transverse, sagittal, and frontal views (**Figure 1**). Rats were positioned in the prone position. Radiation was delivered with a 1.5cm diameter circular collimator that encompassed the left lung and the whole heart. The central axis of the beam (isocenter) was set in the center of the heart, with radiation dose to isocenter of 8 Gy × 5 fractions given once daily, with equally weighted parallel opposed beams. Control rats received anesthesia and sham irradiation. Monte-Carlo-based treatment planning was used to precisely calculate irradiation doses (MAASTRO Radiotherapy Clinic). All rats were maintained in single ventilated cages under pathogen-free conditions at the Biomedical Research Center maintained at a temperature of 23°C on a 12-h:12-h light-dark cycle with access to standard diet (0.4% salt) and water (reverse osmosis hyperchlorinated water).

### Lisinopril

Rats were given the angiotensin converting enzyme inhibitor (ACEi) lisinopril (40 mg/L in the drinking water for an approximate dose of 24 mg m^-2^ day^-1^). Lisinopril was started 7 days after radiation and continued until the experiment was terminated. Seven days after irradiation represents the lower limits of time required for identification and screening of individuals and distribution of countermeasure therapy after a bioterrorism event. It is therefore a treatment window with which we have experience for other radiation associated injuries (48, 49).

### Echocardiography

Transthoracic echocardiography was performed in anesthetized (2% isoflurane) animals at 60, 90 and 120 days after radiation, or at the corresponding time in nonirradiated, age-matched controls. Measurements and data analyses were performed by an investigator blinded to the study groups. Animals were studied in the left lateral decubitus position with a commercially available echocardiographic system (Vivid 7, General Electric, with an 11-MHz M12-L linear array transducer, GE Healthcare, Waukesha, WI). Transthoracic echocardiography was performed from the cardiac short axis of the left ventricle at the papillary muscle level, using the anatomical M-mode feature of the Vivid 7 echo. An M-mode display was generated from raw data 2D images with the line selected passing through the anterior and inferior segments. Stroke volume (SV) was measured using left ventricular end diastolic volume (EDV) and end systolic volume (ESV) using the formula SV = EDV – ESV. Ejection fraction (EF) was measured using the formula EF = SV/EDV × 100 (50). Cardiac output was calculated by multiplying the heart rate × SV. The LV mass was derived from the anteroseptal thickness (AST) and inferolateral thickness (ILT) using the formula: 0.8 (1.04[(ILT + LVIDd + AST)3 − LVIDd3]) + 0.6 (50). Three consecutive heart beats were measured, and the average used for analysis.

### Determination of Blood Bioenergetics in PBMC (Peripheral Blood Mononuclear Cells)

Mitochondrial bioenergetic health can be assessed in circulating platelets and leucocytes, and these values have the potential to be a biomarker for assessing the energetic state of an individual’s vital organs. The Agilent Seahorse XF Cell Mito Stress Test is a standard method for assessing mitochondrial bioenergetic function. Evaluations of multiple metrics of mitochondrial function are derived from oxygen consumption rates measured in the presence of a panel of inhibitors, to extrapolate values for non-mitochondrial respiration, basal respiration, maximal respiration, proton leak, ATP production and spare respiratory capacity. Oxygen consumption rates (OCR) were evaluated in PBMC at ~ 90 days after irradiation. Whole blood was serially harvested from the jugular vein of rats, using EDTA as an anticoagulant. PBMC were isolated by gradient centrifugation. Briefly, 1 ml of whole blood was diluted with 2 ml of Dulbecco’s Phosphate Buffered Saline (Gibco Cat# 14190-144) and layered onto 3 ml of Histopaque (Sigma-Cat# 10831). These tubes were centrifuged for 30 min at room temperature at 400×g. After centrifugation, the plasma layer was removed carefully by aspiration under gentle vacuum. The buffy coat containing the white blood cells was transferred to a 15 ml conical tube with a transfer pipette. The cells were washed with 10 ml of DPBS and centrifuged at 500×g for 10 min at room temperature. The cell pellet was resuspended in 1 ml of RPMI-1640 medium (Life Technologies Cat# 31800-022) supplemented with sodium pyruvate (1 mM). An aliquot was further diluted in RPMI-1640 (1:50 v/v) to determine the cell count. The original cell suspension was then adjusted to 3.25×106 cells per ml with RPMI-1640. Equal numbers of cells were transferred to a 96 well Seahorse XF plate (0.1 ml/well), centrifuged (250×g, 5 min) and additional medium aliquot (80 µl/well) was added. The oxygen consumption rate was measured using a Seahorse XF96 Extracellular Flux Analyzer (Agilent, USA) equipped with the Wave Desktop and Controller 2.6.1 version Software (47, 48). OCR was measured in at least triplicate wells at basal condition and after sequential additions of (i) Oligomycin (complex V-ATP synthase inhibitor, 1 µg/ml), (ii) Carbonyl cyanide-p-trifluoromethoxyphenylhydrazone (FCCP, mitochondrial uncoupler, 1 µM), and (iii) antimycin A (complex III inhibitor, 1 µM) + rotenone (complex I inhibitor, 1 µM). The oligomycin-inhabitable OCR is a measure of the contribution of ATP synthesis to the total OCR. FCCP-induced OCR leads to maximal respiration and enables the determination of the maximum and spare respiratory capacity parameters. Antimycin A + rotenone were added to completely block mitochondrial respiration and determine the contribution of mitochondrial and non-mitochondrial oxygen consuming enzymes to the total OCR values. In addition, the difference between the OCR values after oligomycin and after antimycin A and rotenone injection is a measure of mitochondrial proton leak. The average value for each condition was calculated. After completion of the assays, the medium was aspirated and 20 µl of cell lysis buffer (0.1% Triton X-100, 10 mM Tris-HCl, pH 7.0) was added. The protein content in each well was determined with Bradford reagent (Bio-Rad Cat# 5000002) by measurement of absorbance at 595 nm. The OCR values were expressed as pmoles O_2_/min/µg of protein.

### Statistics

Repeated measures mixed model with the Kenward-Roger approximation (51) was used for restricted maximum likelihood. The model was run separately for each of the following outcomes: EDV in µL/(kg bpm), IVSD in mm, and LVPWD in cm/kg. The factorial treatments of radiation and Lisinopril were estimated adjusting for day (30:intercept, 60, 90 or 120) and strain (BN:intercept, SS and SSBN6) while two-way interactions were investigated. Each experiment included 230 observations. Analyses were run in SAS 9.4, Analytical Products 15.1.

Differences between groups of bioenergetic respiratory parameters in each strain of rats were tested by Analysis of Variance (ANOVA) followed by All Pairwise Multiple Comparison Procedures (Student-Newman Keul’s Method). Student’s t-tests were used in some cases to determine differences between 2 parameters. P values <0.05 were determined to be significant.

## Results

### End Diastolic Volume

**Figure 2:**
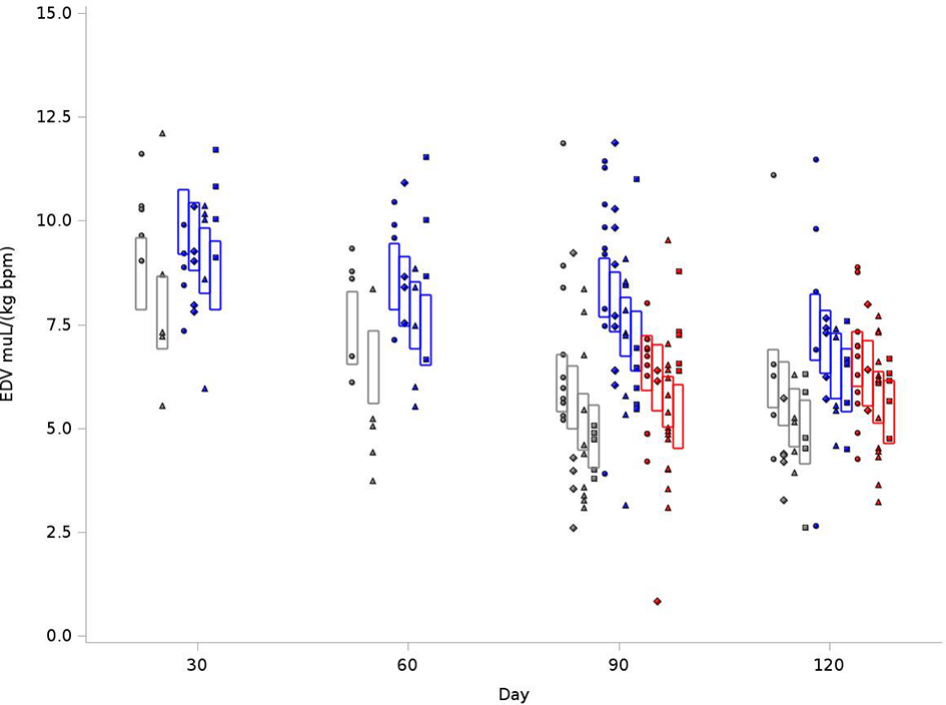
Graphical representation of values for End Diastolic Volumes (EDV) in rats at 4 time points (X-axis). The rats were measured at 4 time points: 30, 60, 90 and 120 days. The values observed are represented by symbols denoting the experimental treatment: Circle (No Radiation and No Lisinopril -R-L), Diamond (No Radiation and Lisinopril -R+L), Triangle (Radiation and No Lisinopril +R-L), or Square (Radiation and Lisinopril +R+L). The colors represent the strain: Brown-Norway (BN: black), Salt-Sensitive (SS: blue) and SS with BN chromosome 6 (SSBN6: red). The rectangles are 95% Confidence Intervals for EDV from a Repeated Measures linear model for treatment that included time, strain, and the SS by day 90 interaction. EDV were normalized to body weight and heart rate and was reduced by 9-11% in all irradiated rats at 120 days (p=0.0013) as compared to their non-irradiated counterparts.

**Figure 3:**
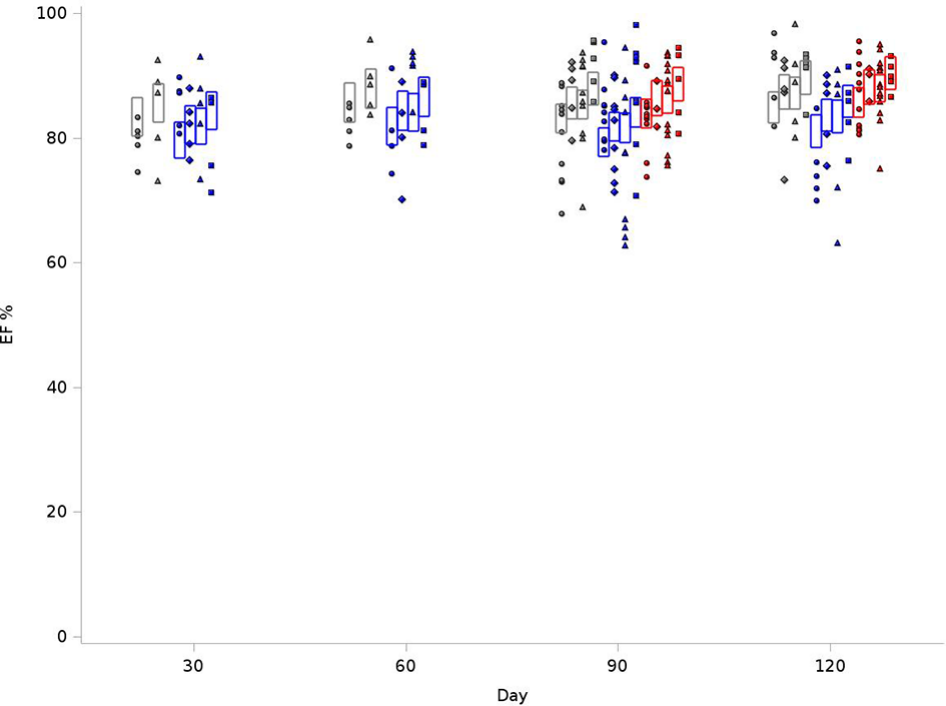
Graphical representation of values for Ejection Fraction (EF) in rats at 4 time points (X-axis). The EFs were measured at 30, 60, 90 and 120 days. The values observed are represented by symbols denoting the experimental treatment: Circle (No Radiation and No Lisinopril -R-L), Diamond (No Radiation and Lisinopril -R+L), Triangle (Radiation and No Lisinopril +R-L), or Square (Radiation and Lisinopril +R+L). The colors represent the strain: Brown-Norway (BN: black), Salt-Sensitive (SS: blue) and SS with BN chromosome 6 (SSBN6: red). The rectangles are 95% Confidence Intervals for EF from a Repeated Measures linear model for treatment that included time and strain. EFs in SS rats were lower than those of other strains (p-value=0.002). They increase with Lisinopril (p-value=0.02) and also with radiotherapy (p-value=0.02).

Because of differences in rat sizes between strains, EDV values were normalized for weight and heart rate. As outlined in **Figure 2**, for the BN control group at 120 days, the average EDV was 8.73 mL/kg(bpm), compared to 9.97 mL/kg(bpm) in SS control and 9.21 mL/kg(bpm) for SSBN6. Radiation reduced end diastolic volume in all rats (-0.94 mL/kg(bpm), p=0.0013), which represents a 9-11% reduction. Lisinopril did not significantly affect the EDV for any of our strains. **Figure 2** demonstrates the changes in EDV values at 30, 60, 90 and 120 days after radiation. Despite the changes in EDV, the EF remained stable across all groups. **Figure 3** shows EFs over time and as a function of treatment group. SS rats had modestly lower EF than other strains, and taking ACE_i_ increases the EF in this strain only. There are, however, no time trends in any group.

### Interventricular Septal Wall Thickness at End Diastole and Posterior Wall Thickness

**Figure 4:**
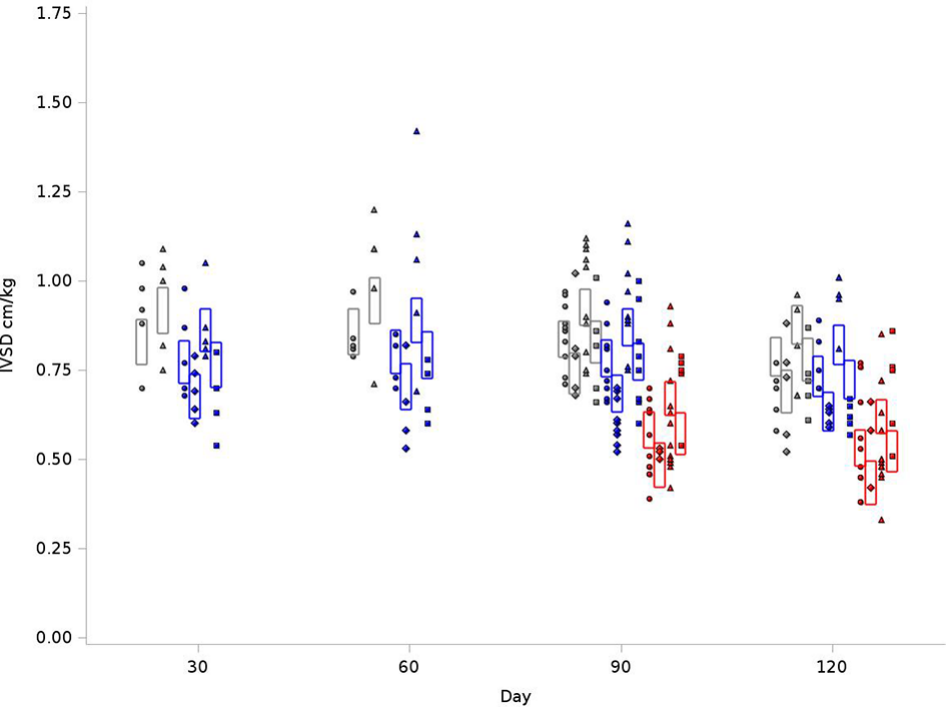
Graphical representation of values for Inter-Ventricular Septum Thickness in Diastole (IVSD) in rats at 4 time points (X-axis). The rats were measured at 4 time points: 30, 60, 90 and 120 days. The values observed are represented by symbols denoting the experimental treatment: Circle (No Radiation and No Lisinopril -R-L), Diamond (No Radiation and Lisinopril -R+L), Triangle (Radiation and No Lisinopril +R-L), or Square (Radiation and Lisinopril +R+L). The colors represent the strain: Brown-Norway (BN: black), Salt-Sensitive (SS: blue) and SS with BN chromosome 6 (SSBN6: red). The rectangles are 95% Confidence Intervals for IVSD from a Repeated Measures linear model for treatment that included time, and strain. IVSD values were normalized to body weight. Radiotherapy increased IVSD by 11-16% at 120 days (p=0.0001) in all rats and lisinopril mitigated this effect by decreasing the IVSD by 12-17% (p < 0.0001).

We examined reduction in chamber size and/or compliance. IVSD values were normalized to body weight. As outlined in **Figure 4**, for the BN control group at 120 days, the average IVSD was 0.83 cm/kg, compared to 0.77 cm/kg in SS control and 0.57cm/kg for SSBN6. Radiotherapy increased the septal wall thickness to 0.92cm/kg, 0.89 cm/kg and 0.66cm/kg, respectively. This represents an 11-16% increase on average in the septal wall thickness for all rats with RT (+0.088 cm/kg, p=0.0001). Lisinopril decreased the IVSD by 12-17% (-0.098 cm/kg, p<0.0001). **Figure 4** demonstrates the changes in IVSD values at 30, 60, 90 and 120 days after radiation.

**Figure 5:**
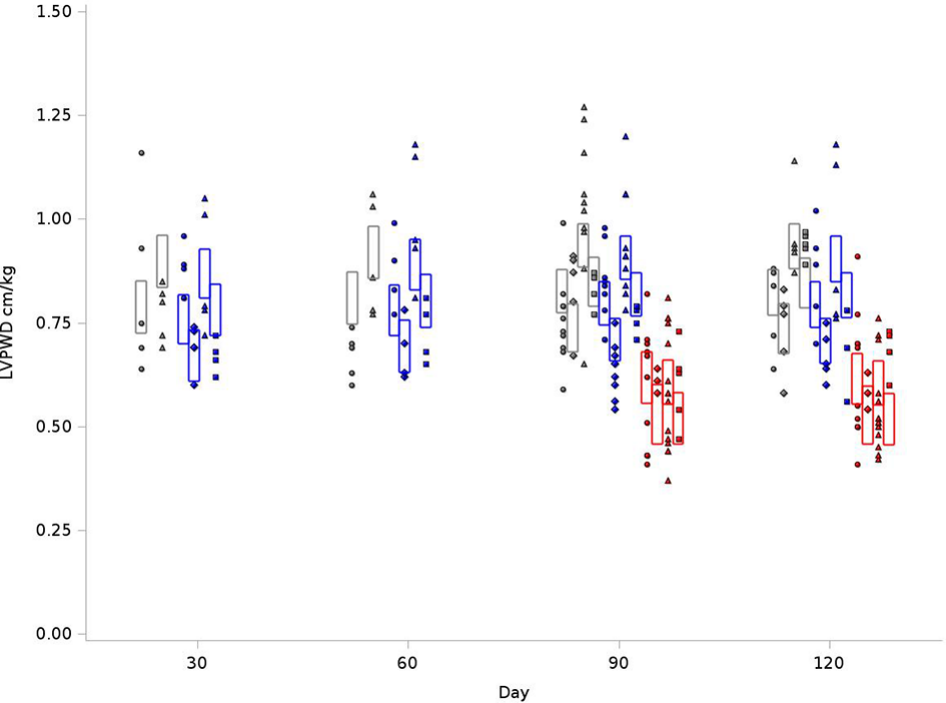
Graphical representation of values for Left Ventricular Posterior Wall Thickness at End Diastole (LVPWD) in rats at 4 time points (X-axis). The rats were measured at 4 time points: 30, 60, 90 and 120 days. The values observed are represented by symbols denoting the experimental treatment: Circle (No Radiation and No Lisinopril -R-L), Diamond (No Radiation and Lisinopril -R+L), Triangle (Radiation and No Lisinopril +R-L), or Square (Radiation and Lisinopril +R+L). The colors represent the strain: Brown-Norway (BN: black), Salt-Sensitive (SS: blue) and SS with BN chromosome 6 (SSBN6: red). The rectangles are 95% Confidence Intervals for LVPWD from a Repeated Measures linear model for treatment that included time, strain and the SSBN6 by radiotherapy interaction. There was 14-15% increase in LVPWD in SS and BN (p<0.0001), but not SSBN6 rat hearts at 120 days, while lisinopril reduced LVPWD in all groups of irradiated rat hearts (p=0.0003).

As outlined in **Figure 5**, for the BN control group, the average LVPWD at 120 days was 0.79 cm/kg, compared to 0.76 cm/kg in SS control and 0.58 cm/kg for SSBN6. There was a 14-15% increase in posterior wall thickness for SS and BN rats who received radiation (+0.11 cm/kg, p<0.0001). For SSBN6 rats, radiation did not increase posterior wall thickness, and in turn demonstrates a decrease of 0.01cm/kg (p=0.0114). Lisinopril reduced LVPWD in all groups, with an average of 11-15% decrease in posterior wall thickness (-0.087 cm/kg, p=0.0003). **Figure 5** demonstrates the changes in LVPWD values at 30, 60, 90 and 120 days after radiation.

### Bioenergetics of Mitochondria in PBMCs

**Figure 6:**
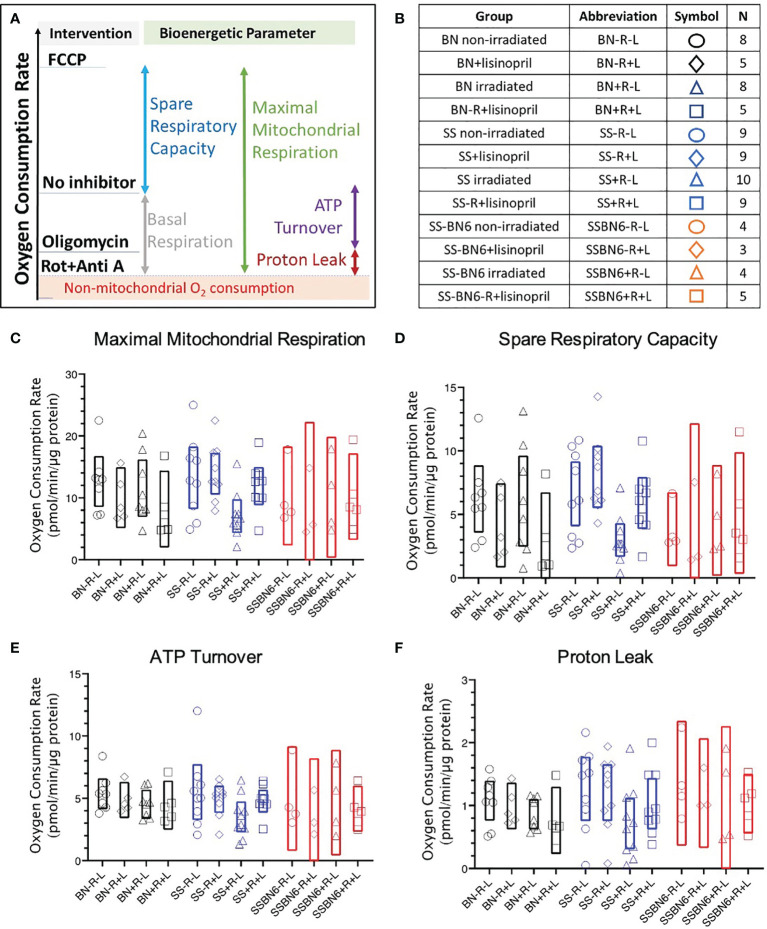
Oxygen consumption rates in peripheral blood mononuclear cells (PBMCs). **(A)** Schematic showing the effects of pharmacological agents on oxygen consumption by PBMCs as investigated by the Seahorse Cell Mitochondrial Stress Test (see *Methods*). If the respiratory chain activity is blocked with Rotenone and Antimycin A (designated as Rot+Anti A) then only non-mitochondrial oxygen consumption remains (shaded and marked in red). The difference between oxygen consumption without an inhibitor and with Rotenone and Antimycin A represents basal mitochondrial respiration (grey bar). Mitochondrial oxygen consumption that is driven by H+ flux through ATP synthase is inhibited by oligomycin. The difference between oxygen consumption without an inhibitor and in the presence of oligomycin gives the oxygen consumption coupled with ATP production (purple bar). The difference between oligomycin-inhibited respiration and non-mitochondrial oxygen consumption gives the proton (H+) leak (maroon bar). The uncoupler FCCP enhances oxygen consumption (blue bar that represents spare respiratory capacity) to yield maximal mitochondrial respiration (green bar). **(B)** Table showing numbers of rats in each group for graphs C-F. Oxygen consumption rates in BN (black), SS (blue) and SSBN6 (red) rats at 90 days post-irradiation. Circles = non-irradiated rats, diamonds = nonirradiated rats given lisinopril, triangles = irradiated rats, squares = irradiated rats given lisinopril. Values are expressed as pmol/minute/microgram protein. **(C)** Maximal mitochondrial respiration. Values were derived as the difference after treatment with the uncoupler FCCP and the non-mitochondrial oxygen consumption rate (represented by green bar in Panel A). FCCP increases the proton flow across the inner mitochondrial membrane creating a H^+^ short circuit to maximize oxygen consumption (p=0.020, SS irradiated rats (SS+R-L) versus SS non-irradiated rats (SS-R-L), p=0.040, SS irradiated rats (SS+R-L) versus SS non-irradiated rats treated with lisinopril (SS-R+L)). There was no difference between other groups. **(D)** Spare Respiratory Capacity derived as the difference between treatment with FCCP and followed by subtraction of the basal oxygen consumption rate in the absence of any inhibitor (represented by blue bar in Panel A), (p= 0.019, SS irradiated rats (SS+R-L) versus SS non-irradiated rats (SS-R-L); p=0.023, SS irradiated rats (SS+R-L) versus SS non-irradiated rats treated with lisinopril (SS-R+L)). There was no difference between other groups. **(E)** ATP turnover after treatment with the ATP synthase inhibitor, oligomycin, and subtraction of the basal oxygen respiration values in the absence of any inhibitor (represented by purple bar in Panel A) (p= 0.037 (t -test, not ANOVA) for SS irradiated rats (SS+R-L) versus SS non-irradiated rats (SS-R-L)). There was no difference between other groups. **(F)** Proton (H+) Leak derived by the difference in oligomycin and the non-mitochondrial oxygen consumption rates (represented by maroon bar in Panel **(A)**. Oligomycin inhibits ATP synthase but not uncoupled mitochondrial oxygen consumption from proton leak (p=0.036 (t -test, not ANOVA), SS irradiated rats (SS+R-L) versus SS non-irradiated rats (SS-R-L)). There was no difference between other groups. Values in rats treated with lisinopril were not different from non-irradiated controls for all mitochondrial respiratory parameters represented in **(E, F)**.

Several mitochondrial bioenergetic parameters (described schematically in **Figure 6A**) were measured in PBMCs harvested from a subset of rats at 3 months post-irradiation (sample sizes shown in **Figure 6B**). The 90 day time point was chosen to predict radiation-induced cardiac dysfunction observed at 120 days. At the start of evaluation, basal respiration was measured without the addition of pharmacological inhibitors. Basal mitochondrial respiration was calculated at the end of the experiments as the difference between the oxygen consumption rate in the absence of inhibitors and after addition of Rotenone with Antimycin A, which shuts down the respiratory chain to ablate mitochondrial oxygen consumption. There was no difference in basal respiration or non-mitochondrial respiration between treatment groups from BN, SS or SSBN6 rats (results not shown).

Maximal respiration was measured by addition of the potent uncoupler FCCP. This stimulated oxygen consumption by uncoupling oxidative phosphorylation and disrupting ATP synthesis to freely permit protons to be transported across cell membranes. By subtracting the non-mitochondrial rate of oxygen consumption from this maximal rate of oxygen consumption, the maximal mitochondrial oxygen consumption was derived. Maximal respiration was decreased in PBMC from irradiated SS rats (SS+R-L) as compared to non-irradiated SS rats (SS-R-L) or irradiated SS rats given lisinopril (SS+R+L) (**Figure 6C**). There was no difference in maximal respiration between the other treatment groups from BN, SS or SSBN6 rats (**Figure 6C**).

The difference between the maximal oxygen consumption rate in the presence of FCCP from the basal respiration without inhibitors yielded the spare respiratory capacity of the mitochondria in the PBMCs from each group. Since there was no difference in basal respiration between groups, results were similar to those for maximal respiration (**Figure 6D**). Once again, PBMCs from irradiated SS rats (SS+R-L) had lower spare respiratory capacity than non-irradiated SS rats (SS-R-L) or irradiated SS rats given lisinopril (SS+R+L) (**Figure 6D**).

ATP turnover was measured after addition of oligomycin to block mitochondrial ATP synthase. The difference between oxygen consumption rates without inhibitors (at the start of the measurements) and after addition of oligomycin determined the rates of oxygen consumption that contributed to ATP production. The results for ATP production are represented graphically in **Figure 6E**. There was no difference between treatment groups in BN, SS or SSBN6 rats when results were examined by ANOVA. However, PBMCs from irradiated SS rats (SS+R-L) had lower respiration that was coupled to ATP production than from non-irradiated SS rats (SS-R-L) if a comparison by t-test was made between groups inside each strain. The proton leak in the mitochondrial membranes of PBMCs from different rat groups were determined by subtracting the non-mitochondrial respiration from respiration after treatment with oligomycin, which inhibited ATP synthase. There was no difference between treatment groups in BN, SS or SSBN6 rats when results were examined by ANOVA. The proton leak was lower in mitochondria from PBMCs of irradiated SS rats (SS+R-L) than those of non-irradiated SS rats (SS-R-L, **Figure 6F**) if a comparison by t-test was made between groups inside each strain.

## Discussion

Small animal models, including rodents, have been used for many decades to study cardiac radiation toxicity, given the physiological similarities that these models have to humans (2, 52, 53). In an effort to understand heritable genetic traits that could modify cardiac radiation sensitivity, Salt-Sensitive (SS) and Brown Norway (BN) strain rats have been used as a cardiac radiation toxicity model (2). A number of different dose and fractionation regimens have been utilized in preclinical studies of RIHD, ranging from large single fractions to more clinically relevant fractionated regimens (2). We used a fractionated regimen consisting of 8 Gy x 5 fractions, similar to prior studies that have utilized fractionated radiation (2, 19, 54) and which have reported histological changes (55, 56).

Looking at the general response of our models to radiation therapy, the cardiac function remained stable, which shows the compensatory mechanisms of the heart. Overall, the systolic function of the animals remained very similar between all 3 strains, which was reflected by the very consistent EF and SV values. The ESV reflects this consistency as the ESV aligns with the changes in EDV. From a contractility and LV function standpoint, the doses of radiation were not overtly cardiotoxic. Nonetheless, radiation induced measurable changes in the hearts of BN, SS and SSBN6 rats. Previous studies by Nabbi et al, 2014 demonstrated cardioprotection in SSBN6 rat hearts that were challenged with ischemia-reperfusion *ex vivo (* 27). Such protection was not observed after *in vivo* radiation injury. Perhaps protection may have occurred at later times than 120 days after radiation, which was the longest duration evaluated in the current study.

From a cardiac structure and remodeling perspective, we did observe changes as evidenced by echocardiogram. Prior studies have shown that even partial heart irradiation can produce left ventricular dilation and increased fibrosis in the myocardium and pericardium (16). The linear LV dimensions and the EDV in our BN control rats were within the normal expected range based on previously published results (2, 57). At later timepoints within BN controls, the EDV is reduced. For SS rats, the control animals have a lower EDV which mildly decreases over time. The SSBN6 rat has an unusually low EDV when compared to their parent strains. Despite these differences, radiation reduced EDV in both resistant and sensitive models for radiation induced cardiotoxicity. Changes in EDV are influenced by preload and LV chamber size. In our data, changes in preload, or diastolic filling time, are not evident, given the consistent heart rates between the groups. Therefore, changes in EDV are going to be reflected by some degree of cardiac hypertrophy and reduction in chamber size or compliance. While evaluating changes in LV chamber size, we found that radiation results in increase in both the septal and posterior wall thickness. These changes would be expected if radiation is invoking cellular damage and fibrosis. The improvement in the linear dimensions (IVSD and LVPWD) with lisinopril reflects the possible anti-remodeling and other actions of this drug to reduce myocyte hypertrophy and fibrosis.

Our results show the effects of radiation to the whole heart, including left posterior wall and interventricular septum hypertrophy and reduction in end diastolic volume. Clinically, patients receiving thoracic RT often receive radiation to only part of the heart, instead of a fairly uniform radiation dose to the whole heart. Preclinical studies which use whole heart irradiation have advanced our knowledge of RIHD, but whole heart radiation might not completely represent the clinical pathophysiology spectrum of RIHD (2, 16, 58–60). Understanding the mechanism of normal tissue radiation injury will help us develop models that more accurately represent the radiation effects observed in patients receiving thoracic irradiation. Furthermore, understanding how to mitigate cardiovascular disease in salt-sensitive populations exposed to radiation has tremendous promise for reducing racial disparities in cancer survivorship between Black and White populations.

This study also examined bioenergetic parameters in PBMCs to determine if these were altered in a manner that would predict cardiac toxicity by radiation. There was no difference in bioenergetics in PBMCs from irradiated versus non-irradiated BN or SSBN6 rats at 90 days, one month before the mild cardiac toxicities were detected in **Figures 2**–**5**. However, mitochondria in irradiated SS rats exhibited lower maximal respiration, and spare respiratory capacity, which was mitigated by lisinopril. ATP turnover and proton leak, though not significant by ANOVA, were lowered by radiation if comparisons were made between all combinations of only 2 groups at a time. Evaluation of the blood cell counts at 90 days in all rats (results not shown) showed no differences in the differential white blood cell counts between irradiated and non-irradiated rats, or irradiated rats given lisinopril. Taken together, these findings warrant further studies into the effects of radiation on the BHI of SS rats and the mitigation by lisinopril. Interestingly, only a partial volume of bone marrow was irradiated in this study, implying that radiotherapy may have a more pronounced effect on mitochondrial respiration in low renin animal models such as SS rats. Bone marrow injury typically recovers by 21 days in irradiated rats after partial body exposures (61) and blood cell counts in the current study were normal at 90 days. Therefore, changes in mitochondrial respiration in these circulating blood cells at 90 days may signal global or abscopal effects of radiation in cells that have not been irradiated. Additionally, there may be a genetic component to such effects since SSBN6 rats appeared more protected from mitochondrial dysfunction than SS rats. Future studies should focus attention on similar data in male rats and on irradiation induced changes in coronary endothelial cells.

## Data Availability Statement

The raw data supporting the conclusions of this article will be made available by the authors, without undue reservation.

## Ethics Statement

The animal study was reviewed and approved by Institutional Animal Care and Use Committee of the Medical College of Wisconsin.

## Author Contributions

Conceptualization, MM, BF, AB, EJ. Methodology, JN, FG, BF, MZ, TG, DV, MM. Formal Analysis, SO, RS, JN, FG, BF, JO, MM. Original Draft Preparation and Writing, SO, JN, MZ, JO, MM. Review and Editing, SO, RS, JN, NL, FG, BF, MZ, TG, DV, AB, JO, EJ, MM. All authors contributed to the article and approved the submitted version.

## Funding

Cancer & Cardiovascular Centers, Medical College of Wisconsin, NIAID U01AI133594 and VA Merit Review Award 1I01BX001681.

## Conflict of Interest

The authors declare that the research was conducted in the absence of any commercial or financial relationships that could be construed as a potential conflict of interest.

## Publisher’s Note

All claims expressed in this article are solely those of the authors and do not necessarily represent those of their affiliated organizations, or those of the publisher, the editors and the reviewers. Any product that may be evaluated in this article, or claim that may be made by its manufacturer, is not guaranteed or endorsed by the publisher.

## Abbreviations

ACEi, Angiotensin Converting Enzyme Inhibitor; ANOVA, Analysis of Variance; AST, Antero Septal Thickness; ATP, Adenosine Tri Phosphate; BHI, Bioenergetic Health Index; BN, Brown Norway; DNA, Deoxy Ribonucleic Acid; DPBS, Dulbecco’s Phosphate Buffered Saline; EDTA, Ethylene Diamine Tetra Acetic acid; EDV, End Diastolic Volume; EF, Ejection Fraction; ESV, End Systolic Volume; FCCP, tri Fluoromethoxy Carbonyl Cyanide Phenylhydrazone; Gy, Unit of Radiation dose; ILT, Infero Lateral Thickness; IVSD, Inter Ventricular Septum Diastole; LV, Left Ventricle; LVIDd, Left Venticular Internal Dimension Diastole; LVPWD, Left Ventricle Posterior Wall Diastole; OCR, Oxygen Consumption Rate; PBMC, Peripheral Blood Mononuclear Cells; RIHD, Radiation Induced Heart Disease; RPMI, 1640 Cell culture medium; SS, Dahl salt-sensitive; SSBN6, Consomic rat with substitution of rat chromosome 6 from BN rat on SS background; SV, Stroke volume.
